# Traumatic life experiences and religiosity in eight countries

**DOI:** 10.1038/s41597-020-0482-y

**Published:** 2020-05-08

**Authors:** Jonathan Jong, Adam Baimel, Robert Ross, Ryan McKay, Matthias Bluemke, Jamin Halberstadt

**Affiliations:** 1 Centre for Trust, Peace, and Social Relations, Coventry University, Coventry, UK; 2 Department of Psychology, Health and Professional Development, Oxford Brookes University, Oxford, UK; 3 Department of Philosophy, Macquarie University, Sydney, Australia; 4 Department of Psychology, Royal Holloway University of London, Egham, UK; 5 GESIS—Leibniz Institute for the Social Sciences, Mannheim, Germany; 6 Department of Psychology, University of Otago, Dunedin, New Zealand

**Keywords:** Human behaviour, Risk factors

## Abstract

We present two datasets from a project about the relationship between traumatic life experiences and religiosity. These include data from 1,754 individuals in the United States (n = 322), Brazil (n = 205), China (n = 202), India (n = 205), Indonesia (n = 205), Russia (n = 205), Thailand (n = 205), and Turkey (n = 205). Surveys were consistent across samples: they include measures of traumatic life experiences, negative affective traits, existential security, life satisfaction, death anxiety, and various religious beliefs, attitudes, and behaviours. Psychometric evaluations of measures of supernatural belief and death anxiety were conducted.

## Abstract (summary)

**Table:** 

Measurement(s)	response to trauma exposure • religiosity
Technology Type(s)	survey method
Factor Type(s)	sex • age • ethnicity • marital status • employment status • socioeconomic status
Sample Characteristic - Organism	Homo sapiens
Sample Characteristic - Location	United States of America • Brazil • China • India • Indonesia • Russia • Thailand • Turkey

Machine-accessible metadata file describing the reported data: 10.6084/m9.figshare.12173592

## Background & Summary

Psychological research on religion is still largely confined to North American and Western European contexts, though the situation is rapidly improving. Starting with the first phase of the International Death Survey (IDS)^1^, our team has been collecting psychometrically evaluable data on various dimensions of religiosity and their correlates from diverse contexts. Expanding the IDS in a new and independent round of data collection, the Negative Experience and Religiosity project retained measures of supernatural belief and death anxiety, but shifted the focus to negative—including potentially traumatic—life experiences, such as unemployment, bereavement, disease, injury, and physical and sexual abuse. This allows researchers to examine relationships between exposure to such negative events, religious beliefs and behaviours, and psychological outcomes. These variables are highly relevant to theories about religion as a coping strategy, both in terms of whether religion is a response to adversity and to whether it offers effective psychological buffers in the face of adversity^2,3^. Indeed, responding directly to Norris and Inglehart’s hypothesis that existential security—“a sense of confidence and predictability in a threatening and uncertain world” (p. 3389)^4^—is conducive to secularism, we included a measure of subjective existential security in this study. Most of the evidence for this hypothesis has come from country-level data, and few studies have attempted to measure existential insecurity subjectively, despite the fact that it is theorised to be a psychological variable for which objective socioeconomic variables are a proxy.

Besides the main variables of interest—exposure to negative life experience and adversity, and religious belief and behaviour—this study also included more specific measures of religiosity, namely measures of “God locus of control” and of “God concepts”. God locus of control refers to the extent to which one believes that things occur at least in part as a function of God’s plans and interventions. God concepts are one’s valanced views about God, which can be positive or negative. These may serve as moderator variables or outcome variables in a theoretical model about the relationship between negative life experiences and religiosity. Finally, two measures of psychological well-being were included—the Depression Anxiety Stress Scales and the Satisfaction with Life Scale—to examine how adversity, religiosity, and psychological well-being interact.

These datasets include data from eight countries, chosen to maximize religious diversity. The USA, Brazil, and Russia are predominantly Christian countries, representing Protestantism (49% in USA in 2017)^5^, Roman Catholicism (65% in Brazil in 2010)^6^, and Eastern Orthodoxy respectively (72% in Russia in 2008)^7^. Indonesia and Turkey are both majority Sunni Muslim countries (>80% in both cases in 2000 and 2006 respectively)^8^. India is a majority Hindu country (79.5% in 2010 based on projections from 2001 census data; Shaivite and Vaishnavite schools are dominant) and Thailand is majority Buddhist (93.2% in 2010 based on projections from 2000 census data; Theravada Buddhism is the dominant school)^9^. Religion in China is notoriously difficult to describe: it has a Buddhist history, combined with elements of Taoism and Confucianism that are sometimes considered religious. Polls generally show that most Chinese self-identify as non-religious (81% in 2000)^10^. These countries were also selected because they are home to a large proportion of their respective dominant religions, not least because of how populous they are. The largest share of Protestants live in the USA (~20%), which also has the largest share of Christians in the world (11.3%); Russia is home to the largest Eastern Orthodox population (~38%), and Brazil to the largest Roman Catholic population (~13%); Indonesia has the largest share of Muslims of any country (~13%); India has over 90% of the world’s Hindu population; Thailand has the second largest share of Buddhists (~13%) after China, which is also home to the largest proportion of religiously unaffiliated people (~62%)^9^.

Given the cross-sectional design of this study, no causal conclusions can be drawn. However, as the study contains theorised causal, mediating, moderating, and outcome variables, it offers the possibility of minimal tests, such as via path analyses, of causal models describing the relationships among these variables^11,12^.

This research was designed in accordance with the regulations of the Research Ethics Committee of Royal Holloway, University of London, and has been reviewed and approved by these bodies (Ref. 2015/102).

## Methods

The survey—which can be downloaded in full from the Open Science Framework^13^—contains seven sections, always presented in the following order:

1. **Demographics**. We collected data on gender, age, ethnicity, marital status, employment status/occupation, and socioeconomic status.
2. **Traumatic Life Experiences Inventory**. We included a 29-item measure of traumatic life experiences—the Traumatic Life Experiences Inventory—adapted from previous batteries^14,15^. Participants indicated whether or not each of the events has ever occurred to them.
3. **Depression Anxiety Stress Scales**. Lovibond and Lovibond’s 21-item Depression Anxiety Stress Scales (DASS-21) was used to measure negative affective traits^16^. The DASS-21 is widely used and has been validated in multiple studies across multiple cultural contexts. Psychometric evaluations of the DASS-21 have been positive, with the caveat that its subscales—depression, anxiety, and stress—may not be as distinct as originally theorised^17–20^.
4. **Subjective Existential Security**. Norris and Inglehart have observed that there is a paucity of subjective measures of existential security^4^. Therefore, we developed a simple 6-item measure that asks participants about how much they worry about financial matters, health, personal safety, personal relationships, and social status.
5. **Religiosity**. A variety of religiosity measures were included. The first two items pertained to participants’ upbringing; this was followed by items asking about their current self-description as religious and spiritual, and their specific religious affiliation/s. We then included Jong and Halberstadt’s 6-item Supernatural Belief Scale^1,21^. Next came a 5-item measure of religious behaviour, developed in our previous work. We also included Welton, Adkins, Ingle, and Dixon’s^22^ 6-item “God control” supplemental subscale of Levenson’s multidimensional locus of control measure^23^. Finally came Shariff and Norenzayan’s 14-item Views of God scale^24^ that asked about whether positive (e.g., forgiving, loving) and negative (e.g., vengeful, fearsome) traits applied to God.
6. **Satisfaction with Life**. As a contrast to the subjective existential security measure, we also included Deiner, Emmons, Larsen, and Griffin’s widely used and previously validated Satisfaction with Life Scale (SWLS)^25^. Like the DASS-21 above, the SWLS is well-regarded and has enjoyed extensive cross-cultural use^26^.
7. **Death anxiety**. Two measures of death anxiety were included: Conte, Weiner, and Plutchik’s Death Anxiety Questionnaire (DAQ)^27^ and Jong, and Halberstadt’s Existential Death Anxiety Scale (EDAS)^1,21^. The order of these two scales was counter-balanced across participants.

The survey, originally composed in English, was translated by Qualtrics’s partner Language Connect into Brazilian Portuguese, Chinese (Simplified), Hindi, Tamil, Bahasa Indonesia, Russian, Thai, and Turkish. The translation process involved an initial translation, followed by a check by the same translator; the resulting translation was then independently checked again before being uploaded to Qualtrics. Qualtrics Panels then recruited participants in December 2015 and January 2016 from the USA, Brazil, China, India, Indonesia, Russia, Thailand, and Turkey. The target sample size was n = 200 for each country, except the USA (n = 300). To obtain these samples, Qualtrics Panels collected 1,816 responses. Of these, 59 were excluded as they did not fully provide informed consent; a further 3 were excluded due to missing explicit location (i.e., country) information. This resulted in 1,754 completed surveys.

Several checks were put in place to increase data reliability. A two-stage attention check was included after the Traumatic Life Experiences Inventory. It was disguised as a Positive and Negative Affect Schedule^28^ (PANAS): instead of selecting options to indicate their current emotional state, participants were asked to only check the “none of the above” option. If they failed to comply, they were informed that the PANAS was an attention check, asked to try again, and encouraged to pay closer attention to the rest of the survey. Of the 1,754, 935 participants passed the first time, and were automatically retained; 815 participants failed the first time but passed the second time, and were also retained; 4 participants failed twice, and were rejected.

At the end of the survey, participants were asked whether they responded randomly or provided responses that were false at any point during the survey. 1,668 indicated that they did not, 86 indicated that they did. For the purposes of our main analyses, both groups of participants were retained.

Aggregating across countries, approximately 55% of the participants were male and 45% were female: males were over-represented in India, Indonesia, and Turkey. The sample comprised approximately 28% Christians, 19% Muslims, 13% Buddhists, 9% Hindus, 25% nonreligious/atheist/agnostic, and 6% other (see Table 1). For comparison, Pew’s estimate of the global distribution of religions in 2020 is 31.1% Christian, 24.9% Muslim, 6.6% Buddhist, 15.2% Hindu, 15.6% unaffiliated, and 6.6% other^29^. An oversampling of nonreligious individuals led to proportions of the dominant religious group in each country being lower than Pew’s 2020 projections, especially in the United States, where our largest religious group was  none/atheist/agnostic at 49% with only 40% of participants identifying as Christian (Pew: 18.6% and 75.5% respectively). Our samples matched Pew’s estimates more closely in most other countries: 70% Christian in Brazil (Pew: 88.1%), 73% Hindu in India (78.9%), 68% Muslim in Indonesia (87%), 70% Christian in Russia (72.9%), 87% Buddhist in Thailand (92.6%), and 81% Muslim in Turkey (98%). The exception to this trend was China, where the nonreligious were the dominant category in our sample (71%) as well as Pew’s estimate (51.8%).

**Table 1:** Gender and age demographic information by country.

Country	Female	Male	Other	Age range	Age M (SD)	Dominant religious group(s) (%)
Brazil	121	84	0	18–64	36.1 (11.6)	Christian (69.3)
China	102	100	0	18–67	35.2 (9.07)	None/Atheist/Agnostic (71.3)
India	66	139	0	18–75	31.6 (9.38)	Hindu (72.7)
Indonesia	72	133	0	18–63	32.8 (8.88)	Muslim (68.3)
Russia	115	90	0	17–58	34.2 (9.59)	Christian (69.8)
Thailand	94	108	3	17–68	34.7 (10.7)	Buddhist (86.8)
Turkey	68	136	1	18–91	33.1 (11.0)	Muslim (80.5)
USA	150	169	3	19–70	36.0 (11.5)	None (48.8), Christian (39.8)

## Data Records

All three datasets have been anonymised and are available in XLSX and CSV (non-proprietary) formats on the Open Science Framework (OSF) platform^13^ together with files of the questionnaires. Abbreviation guides for variable names are also included in each XSLX file as well as in separate CSV files.

## Technical Validation

As psychometric information has previously been published about the EDAS and SBS-6 used in the USA, Brazil, and Russia under similar recruitment conditions, we compared our results to those previous results^1^. As shown in Table 2, Guttman split-half reliability (λ_4_) for both SBS and EDAS were similar in our study as compared to the previous study. We also ran principal axis factoring on both the SBS and EDAS to extract a single factor. The proportion of variance explained by the one factor solution varied from country to country, but was always >50%. Furthermore, the proportion of variance was similar in our study as in the previous study, with the exception of the SBS in Russia: the first factor now explains less variance than before.

**Table 2:** SBS and EDAS split-half reliability and % explained by single factor [with 2019 study results].

Country	EDAS, λ_4_ [2019]	EDAS, % variance	SBS, λ_4_	SBS, % variance
Brazil	0.99 [0.95]	0.78 [0.74]	0.93 [0.92]	0.51 [0.62]
China	0.99	0.84	0.94	0.68
India	0.99	0.77	0.92	0.60
Indonesia	0.98	0.73	0.91	0.53
Russia	0.98 [0.92]	0.74 [0.71]	0.96 [0.88]	0.72 [0.58]
Thailand	0.99	0.79	0.94	0.58
Turkey	0.98	0.70	0.97	0.79
USA	0.99 [0.96]	0.83 [0.83]	0.98 [0.96]	0.85 [0.86]

As this dataset also included other measures of death anxiety and religiosity, we were able to run tests of convergent validity. As the same measures appeared in the previous dataset in the USA, Brazil, and Russia, we were able to compare our findings with previous results in these countries. As before, SBS scores were correlated with other measures of religiosity: furthermore, both in this dataset and in the previous study, the correlation between SBS scores and religious volunteerism stood out as very weak in Russia (see Table 3). This is likely because in both studies, self-reported levels of religious volunteerism in Russia were low. EDAS was correlated with DAQ in every country, and effect sizes were similar between this study and previous research (see Table 4).

**Table 3:** SBS correlations with related variables [with 2019 study results].

Country	Religious	Spiritual	Pray privately	Attend services	Visit sites or shrines	Volunteer	Tell others
Brazil	0.50 [0.55]	0.57 [0.48]	0.55 [0.62]	0.37 [0.44]	0.36 [0.44]	0.20 [0.32]	0.42 [0.42]
China	0.63	0.34	0.57	0.52	0.37	0.43	0.56
India	0.54	0.49	0.59	0.58	0.54	0.48	0.47
Indonesia	0.43	0.36	0.52	0.41	0.37	0.27	0.33
Russia	0.64 [0.54]	0.35 [0.35]	0.45 [0.48]	0.40 [0.38]	0.38 [0.32]	0.10* [*0.09*]	0.21 [0.28]
Thailand	0.42	0.63	0.45	0.30	0.35	0.30	0.39
Turkey	0.70	0.56	0.63	0.58	0.47	0.36	0.39
USA	0.70 [0.77]	0.78 [0.81]	0.73 [0.79]	0.47 [0.58]	0.35 [0.42]	0.39 [0.46]	0.49 [0.54]

*n.s*.

*p < 0.05.

Else, p < 0.001.

**Table 4:** EDAS correlation with DAQ [with 2019 study results].

Country	*r*(EDAS, DAQ) [2019]
Brazil	0.75 [0.78]
China	0.69
India	0.77
Indonesia	0.78
Russia	0.64 [0.65]
Thailand	0.67
Turkey	0.71
USA	0.71 [0.77]

Taken together, our internal reliability analyses, factor analyses, and convergent validity analyses indicate that the data are sound. However, as there are ways to apply more stringent exclusion criteria to our data, we compared two subsets for each country: the first subset included only participants who passed the attention check the first time and indicated that they did not respond randomly or falsely (“Pass”), whereas the second subset included participants who failed the first attention check (but passed the second) and/or indicated that they responded randomly or falsely (“Fail”). Aggregating across all countries, this method of division produced approximately equally sized groups (48% Fail): however, subset sizes differed from country to country. Notably, very few participants from the USA failed even on these stringent criteria (6.2%): between-group comparisons for the USA are therefore unreliable. As mentioned above, only 86 participants across all countries indicated that they had responded randomly or falsely: 67% of those who failed here failed the attention check the first time, and all but one passed the second time.

**Fig. 1:**
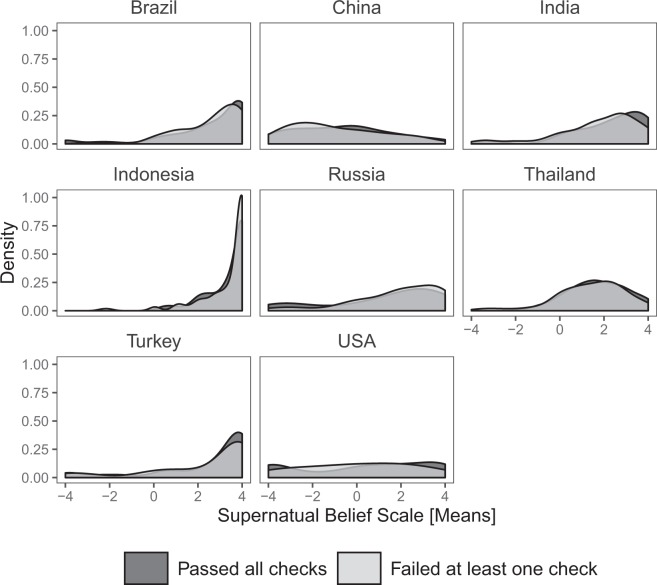
Density plot for SBS by country and performance on attention and/or honesty check.

**Fig. 2:**
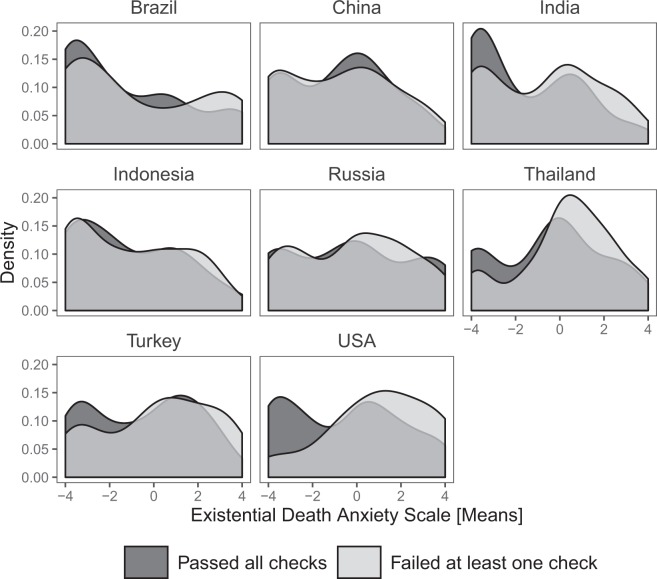
Density plot for EDAS by country and performance on attention and/or honesty check.

**Fig. 3:**
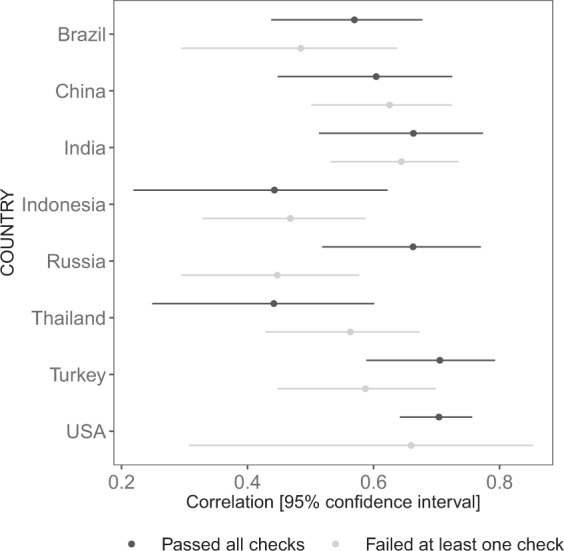
Correlations between SBS and religiosity index.

**Fig. 4:**
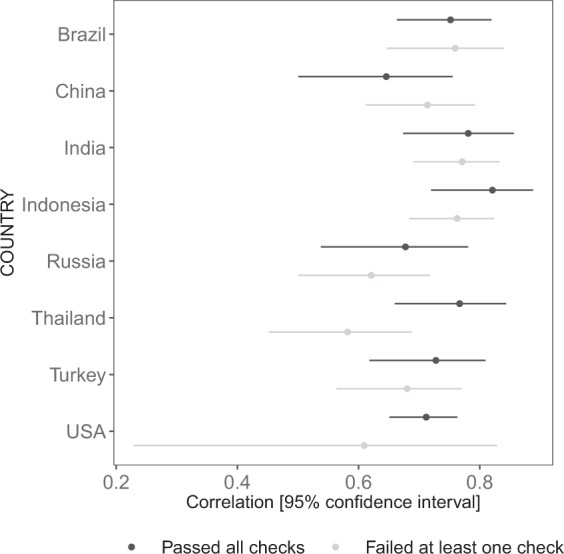
Correlations between EDAS and DAQ.

As shown in Table 5 and Fig. 1, the central tendencies and distribution of values for the SBS are similar between the two subsets in most countries. T-tests found a significant difference only in India, though the magnitude of difference was similar in Russia. Differences were more marked for the EDAS: t-tests found significant differences in India, Thailand, and Turkey (Table 6). However, the density plots show that distributions were similar between subsets for all countries (Fig. 2). None of the significant tests were corrected for multiple comparisons. Finally, we inspected correlations between SBS and other religiosity measures, and between EDAS and DAQ. For the former analyses, we correlated SBS with the mean of the seven other religiosity items (cf. Table 3). As shown in Figs. 3 and 4, the magnitudes of the correlations were similar between the subsets in each country: 95% confidence intervals overlapped in every case. The confidence intervals for the USA are large due to small sample size, as very few participants failed our data quality checks.

**Table 5:** Descriptive statistics for SBS by country and performance on attention and/or honesty check.

Country (n_pass_, n_fail_)	Total, Mean (SD)	Pass, Mean (SD)	Fail, Mean (SD)	Δ_pass-fail_
Brazil (125, 79)	2.54 (1.80)	2.50 (1.99)	2.60 (1.47)	−0.103
China (84, 118)	−0.754 (2.14)	−0.607 (2.20)	−0.859 (2.10)	0.252
India (75, 130)	1.96 (1.80)	2.30 (1.69)	1.76 (1.84)	0.543*
Indonesia (63, 142)	3.35 (1.07)	3.37 (0.93)	3.35 (1.13)	0.024
Russia (80, 125)	1.57 (2.25)	1.21 (2.55)	1.79 (2.02)	−0.576
Thailand (82, 123)	1.54 (1.46)	1.75 (1.30)	1.41 (1.56)	0.341
Turkey (97, 108)	2.38 (2.35)	2.45 (2.32)	2.32 (2.38)	0.124
USA (302, 20)	0.32 (2.86)	0.341 (2.89)	0.10 (2.55)	0.241

*p < 0.05.

Else, n.s.

**Table 6:** Descriptive statistics for EDAS by country and performance on attention and/or honesty check.

Country	Total, Mean (SD)	Pass, Mean (SD)	Fail, Mean (SD)	Δ_pass-fail_
Brazil	−1.02 (2.79)	−1.20 (2.70)	−0.072 (2.93)	−0.495
China	−0.720 (2.35)	−0.719 (2.28)	−0.720 (2.40)	0.001
India	−0.951 (2.47)	−1.49 (2.38)	−0.640 (2.48)	−0.850*
Indonesia	−1.04 (2.40)	−1.15 (2.38)	0.995 (2.41)	−0.158
Russia	−0.184 (2.58)	−0.226 (2.74)	−0.157 (2.48)	−0.07
Thailand	0.0195 (2.30)	−0.437 (2.45)	0.324 (2.16)	−0.761*
Turkey	−0.118 (2.46)	−0.491 (2.39)	0.217 (2.48)	−0.707*
USA	−0.443 (2.56)	−0.54 (2.55)	1.01 (2.34)	−1.55

*p < 0.05.

Else, n.s.

In summary, in the full samples for each country, the two key measures—EDAS and SBS-6—behaved as expected, consistent with previous research, demonstrating high levels of internal reliability and convergent validity with related indicators. Applying a more stringent exclusion criterion made minimal difference to the distribution of values of the SBS and EDAS, though some differences in central tendencies were detected. Correlations between these measures and related measures were similar between Pass/Fail subsets in all countries.

## Data Availability

An R script and wrapper for the data analyses presented here are available as.rmd and.rproj files on the Open Science Framework (OSF) platform together with the questionnaires and data files^13^. These can be used to generate exploratory factor analyses and estimates of split-half reliabilities for the focal scales (SBS and the EDAS) independently in each country. All data presented in tables and figures in the manuscript can be easily reproduced using the provided code. Those interested in following up on our analyses can use our base-script as a starting point.
